# Valorisation of Red Gypsum Waste in Polypropylene Composites for Agricultural Applications

**DOI:** 10.3390/polym17131821

**Published:** 2025-06-30

**Authors:** Chiara Pedrotti, Damiano Rossi, Marco Sandroni, Irene Anguillesi, Chiara Riccardi, Pietro Leandri, Miriam Cappello, Sara Filippi, Patrizia Cinelli, Massimo Losa, Maurizia Seggiani

**Affiliations:** Dipartimento di Ingegneria Civile ed Industriale (DICI), Università di Pisa, Largo Lucio Lazzarino 1, 56122 Pisa, Italy; chiara.pedrotti@ing.unipi.it (C.P.); maurizia.seggiani@unipi.it (M.S.)

**Keywords:** red gypsum, calcium sulfate, polypropylene, stearic acid, composites, recycling, solid waste

## Abstract

This study investigates the industrial potential of red gypsum (RG), a major by-product of titanium dioxide (TiO_2_) production, for the development of thermoplastic polypropylene (PP)-based composites via melt extrusion, targeting agricultural applications. Prior to compounding, RG was thermally treated at approximately 200 °C to remove residual moisture and chemically bound water, resulting in its anhydrous form (CaSO_4_). PP/RG composites were then formulated with RG loadings up to 20 wt.%, employing stearic acid (SA) as a compatibilizer. The resulting materials were thoroughly characterized and successfully processed through industrial-scale injection molding up to 250 °C. Morphological and FTIR analyses confirmed the role of SA in enhancing both filler dispersion and interfacial adhesion between RG and the PP matrix. SEM images revealed finer and more uniformly distributed RG particles, resulting in a reduced loss of ductility and elongation at break typically associated with filler addition. Specifically, the Young’s Modulus increased from 1.62 GPa (neat PP) up to 3.21 GPa with 20 wt.% RG and 0.6 wt.% SA. The addition of 0.6 wt.% SA also helped limit the reduction in stress at break from 46.68 MPa (neat PP) to 34.05 MPa and similarly mitigated the decrease in Charpy impact energy, which declined slightly from 2.66 kJ/m^2^ (neat PP) to 2.24 kJ/m^2^ for composites containing 20 wt.% RG. Preliminary phytotoxicity was assessed using germination tests on *Lepidium sativum* L. seeds. Eluates from both untreated and SA-treated RG powders resulted in germination indices below 80%, indicating phytotoxicity likely due to high sulfate ion concentrations. In contrast, eluates from composite pellets exhibited germination indices equal to or exceeding 100%, demonstrating the absence of phytotoxic effects. These results highlight the suitability of the developed composites for applications in floriculture and horticulture. The optimized composite pellets were successfully processed via injection molding to manufacture plant pots, which exhibited a dark brown coloration, confirming the effective pigmenting function of RG. These results demonstrate the potential of red gypsum to serve both as a functional filler and pigment in PP composites, providing a sustainable alternative to iron oxide pigments and promoting the valorization of industrial waste through resource recovery.

## 1. Introduction

Red Gypsum (RG) is an industrial waste formed from the sulfate process used in the production of titanium dioxide (TiO_2_), from ilmenite (FeTiO_3_) or other titanium-containing ores. The mineral is digested with sulfuric acid (H_2_SO_4_) to form titanium sulfate (TiOSO_4_), which is then calcined to produce titanium dioxide (TiO_2_). The remaining acidic solution is neutralized using calcium carbonate (CaCO_3_) powder or lime slurry, resulting in the formation of large quantities of gypsum-rich sludge. This by-product, commonly referred to as red gypsum due to its reddish coloration from iron hydroxides, is primarily composed of calcium sulfate dihydrate (CaSO_4_·2H_2_O), along with TiO_2_, silica, iron and aluminum oxides, and trace amounts of metal impurities such as Mn, Ni, Cu, Cr, and V, originating from the ilmenite ore. A key factor underscoring both the potential and the necessity for reuse strategies is the substantial volume of red gypsum generated annually: approximately 6 to 10 tons of RG are produced for every ton of TiO_2_ obtained [1,2].

RG is currently classified as non-hazardous special waste under the European Waste Catalogue (EWC) code 06.11.01, which refers to “waste from calcium-based reactions in the production of titanium dioxide” [3]. Although classified as non-hazardous, landfill disposal or storage in controlled industrial waste repositories are the most common methods for managing red gypsum, posing potential risks of groundwater contamination. These practices represent both an environmental concern and a financial burden for titanium dioxide producers. The challenges arise from composition variability, the presence of trace impurities and metals, as well as transportation and processing costs, compounded by restrictive regulations or a lack of clear standards for reuse. Despite these obstacles, the large volumes produced have sparked growing interest of TiO_2_ producers in finding economically and environmentally sustainable recycling and valorization methods for this waste.

One promising approach is its use in the construction sector, particularly as a partial substitute for cement clinkers. However, several limitations hinder its suitability for producing construction-grade materials, including high moisture content, the presence of metallic impurities, fine crystalline morphology, and low mechanical strength [4,5]. RG has also shown potential for agronomic applications, owing to its content of various chemical elements that can serve as nutrients, soil conditioners, and pH stabilizers in acidic soils [6]. In addition, it may contribute to reducing heavy metal leaching and enhancing carbon dioxide sequestration [7]. Ongoing research is exploring further RG valorization routes, such as the extraction of valuable metals (Ti, Fe, V) and the recovery of sulfur [8]. Despite these efforts, the overall recycling rate remains low, typically between 5 and 10%, and a dedicated, well-established supply chain for RG valorization is still lacking [1,2].

In contrast to other fillers [9,10,11,12], the potential of red gypsum as a filler in polymer-based materials remains largely unexplored. This gap presents an opportunity for research into sustainable and value-added uses of RG in composites. Calcium carbonate (CaCO_3_) is the most used calcium-based inorganic filler in composites made of PVC, PE, PP, EVA, and various rubbers, owing to its versatility, relatively low cost, and ease of processing. In contrast, calcium sulfate (CaSO_4_) is less frequently employed due to its higher hygroscopicity and cost. Nonetheless, red gypsum—particularly in its thermally stable, anhydrous form (anhydrite)—can represent a promising alternative filler for polymer matrices such as PLA [13], PP [14], and PVC [15]. Recently, Wang et al. (2025) demonstrated that anhydrite whiskers derived from red gypsum improved the elongation at break, flexural strength, and impact strength of PVC/RG composites [16]. To the best of our knowledge, this study constitutes the only one to date that specifically addresses the use of RG as a filler in the development of polymer-based composites.

Within this context, the present study aims to broaden the applicability of RG as both a filler and a pigment in widely used polymer matrices. Polypropylene (PP) was selected as a matrix to develop PP/RG composites. To enhance the dispersion of RG within the PP matrix, stearic acid (SA) was employed as a green compatibilizer. This choice is based on the chemical nature of common inorganic fillers like CaSO_4_, whose surfaces are rich in hydroxyl groups. SA, featuring a hydrophilic head that bonds to the filler and a long hydrophobic tail that interacts with the polymer, promoting improved filler dispersion and interfacial compatibility. As a result, this surface modification contributes to better interfacial adhesion, enhanced mechanical properties, and improved processing stability in the resulting composites [13,14,16].

PP/RG composites were prepared with different thermally pretreated RG loadings (10 and 20 wt.%) both with and without SA by melt extrusion compounding. The composites were characterized by thermogravimetric analysis (TGA), differential scanning calorimetry (DSC), Fourier Transform Infrared Spectroscopy (FTIR), and mechanical tests (tensile and impact resistance) to evaluate the effects of RG content and of SA addition on processability, thermal stability, crystallinity, and mechanical performance of the PP matrix.

The optimized composite was subsequently processed by injection molding to fabricate polypropylene (PP)-based plant pots, a product widely employed in agriculture and plasticulture [17]. The incorporation of inert, low-cost fillers—such as calcium carbonate (CaCO_3_), fly ash, talc, and clay minerals—is a well-established approach for reducing the consumption of petroleum-derived polymers, including PP, polyethylene (PE), and polyvinyl chloride (PVC), in agricultural applications. Within this framework, RG could serve as an alternative inorganic filler to lower plastic usage and material costs, while enhancing dimensional stability, reducing processing shrinkage, increasing stiffness, improving thermal resistance, and enabling a sustainable route for the valorization of a non-hazardous waste currently lacking a dedicated recycling pathway. In view of this, a preliminary phytotoxicity test was conducted based on the germination index of *Lepidium sativum* L. seeds, using eluates obtained from RG and PP/RG composites.

## 2. Materials and Methods

### 2.1. Materials

A filter-pressed red gypsum obtained from the TiO_2_ production plant operated by Venator in Scarlino (Grosseto, Italy) was used in this study [18].

Table 1 reports the concentrations of major elements (determined by XRF) and trace elements (determined by ICP-MS), expressed as oxides. As shown, the main components are CaO (30.2 wt.%) and SO_3_ (40.8 wt.%), similar to those found in natural gypsum, mixed with TiO_2_ (1.47 wt.%), Fe_2_O_3_, and various other metals, including manganese, nickel, copper, iron, chromium, and vanadium. Additionally, chlorides are also present. According to the analysis, red gypsum mainly consists of CaSO_4_·2H_2_O, accounting for 19.4 wt.% of bound water.

Polypropylene (PP) granules, Moplen HP500N^®^, a polypropylene homopolymer manufactured by LyondellBasell (Houston, TX, USA), were used as a polymer matrix for red gypsum composite production. This polymer grade is specifically designed for general-purpose injection molding and offers a well-balanced combination of excellent flow characteristics and high stiffness. Moplen HP500N^®^ complies with food contact regulations, making it suitable for use in the food packaging industry. Table 2 reports the main properties from the technical data sheet [19].

Stearic Acid (SA) produced by ACEF Spa (Italy) was used to reduce hygroscopicity and enhance the dispersion of red gypsum within the polymeric matrix. SA has a melting point above 53 °C, density of 0.8–0.9 g/cm^3^, pH < 7 and cinematic viscosity at 70 °C of 12 mm^2^/s [20].

### 2.2. Preparation of Blends

#### 2.2.1. Material Pretreatment

Prior to extrusion with PP, the as-received red gypsum was finely ground and sieved using a 150 µm mesh, then dried at 105 °C for 24 h to remove moisture. Subsequently, the dried gypsum was heated at 200 °C for 24 h to remove the chemically bound water and obtain anhydrite (CaSO_4_). Hereafter, the label RG refers to the thermally treated red gypsum.

To improve the processing of RG with PP and enhance its dispersion within the polymer matrix, RG was treated with 3 wt.% SA relative to the anhydrite content. Indeed, Wang et al. (2025) [16], reported that the addition of a small amount of stearic acid—up to 4 wt.%—promotes the formation of a hydrophobic film that ensures complete coverage of the filler particles and improves their compatibility with PP, thereby reducing the risk of RG particle agglomeration.

SA was mixed with RG using a high-speed knife blender. During blending, the temperature reached approximately 70–80 °C, allowing the SA to become fluid and effectively coat the RG powder. Hereafter, the label SA-treated RG refers to the RG coated with red gypsum.

Prior to use, PP granules were also dried at 80 °C for 24 h.

#### 2.2.2. Composite Production

Composite preparation involved the use of trays containing RG powder—with or without SA—and dried PP pellets, combined according to the weight ratios specified in Table 3. The components were manually mixed to ensure uniform distribution of the RG powder among the pellets. The resulting mixture was subsequently processed using a single-screw laboratory extruder (Brabender Plasti-Corder Lab-Station, Duisburg, Germany), equipped with a gravimetric side feeder and operated without mixing elements. The extruder was fitted with a screw of 19 mm diameter, a nominal length of 25 L/D, an axle gauge of 16.5 mm, and a flight depth of 3.75 mm.

The extruder is equipped with five independently regulated heating zones (Table 4). Extrusions were at a screw speed of 60 rpm for neat PP, and between 80 and 100 rpm for the composites. Different screw speeds were employed during the extrusion of PP and PP/RG composites to maintain relatively uniform mechanical and thermal stress across the various samples (torque: 16 ± 2.8 Nm; mass flow rate: 2.5 ± 0.3 kg/h). This approach was necessary to ensure the production of comparable composites under consistent processing conditions. The adjustment was required due to significant variations in interfacial interactions and melt viscosity between the polymer matrix and the composites obtained at different RG and SA concentrations.

After extrusion, the composite filament was cooled in a water bath and pelletized using a Procut 3D variable-speed cutter (Chinchio Sergio Srl, Brescia, Italy). The resultant pellets were dried at 80 °C for at least 24 h and stored in vacuum-sealed bags prior to analysis and the subsequent injection molding.

#### 2.2.3. Lab Scale Injection Molding

Dog-bone (type 1BA) and Charpy specimens (80 mm × 10 mm × 4 mm) were produced by injection molding using a press Megatech H22/50 (Tecnica Duebi Srl, Ancona, Italy), following the thermal profile reported in Table 5. The press operated at a rotational speed of 100 rpm and a maximum injection velocity of 80%, with a back pressure of 3 bar and a final pressure ranging from 100 to 120 bar. The processing time was approximately 20 s.

### 2.3. Characterizations

The morphology of the samples was examined by scanning electron microscopy (SEM) with an EM-30N COXEM microscope (Daejeon, Republic of Korea), operated at voltages ranging from 1 to 30 kV (1 kV increments). Dog-bone specimens were fractured in liquid nitrogen to preserve the fracture surface morphology and assess filler–matrix interfacial adhesion. To enable electrical conductivity, the fractured surfaces were sputter-coated with a uniform gold layer (5–6 nm thick) using an Edwards S150B coater (Burgess Hill, UK).

Chemical interactions between the filler and the PP matrix were investigated through Fourier Transform Infrared Spectroscopy (FTIR) in ATR mode. Spectra were collected using a Cary 630 FTIR spectrometer (Agilent, Santa Clara, CA, USA) over a wavenumber range of 4000–450 cm^−1^, at a resolution of 4 cm^−1^ with 32 scans.

The thermal behavior of the composites was assessed using thermogravimetric analysis (TGA), performed with a Netzsch STA 2500 Regulus (Selb, Germany). Measurements carried out from room temperature to 800 °C at a heating rate of 10 °C/min under a nitrogen atmosphere, with a purge flow of 50 mL/min and a furnace flow of 20 mL/min. The parameters evaluated included: onset degradation temperature (T_onset_), defined as the extrapolated temperature at the start of weight loss; degradation temperature (T_d_), corresponding to the peak of the DTG curve; residual mass percentage and total weight loss.

Additional thermal properties were determined by differential scanning calorimetry (DSC), carried out with a Pyris 1 DSC 6000 (PerkinElmer, Shelton, CT, USA). Samples were analyzed from −60 °C to 200 °C at a heating rate of 10 °C/min under a nitrogen flow of 20 mL/min. The thermal parameters evaluated included the melting enthalpy (ΔH_m_), glass transition temperature (T_g_), melting temperature (T_m_), and degree of PP crystallinity (Χ_C_), calculated as follows (Equation (1)):

(1) $${Χ}_{C}\left(\%\right)=\frac{{\Delta H}_{m\left(PP\right)}}{{\Delta H}_{m\left(PP\right)}^{{}^{\circ}}\cdot f_{PP}}\cdot 100$$

where ΔH°_m_ is the melting enthalpy of 100% crystalline PP (207 J/g [21]) and f_pp_ is the weight fraction of PP in the composite. For a more accurate evaluation of these parameters, only the second heating DSC curve was considered, as it is more representative of the intrinsic material behavior.

Tensile tests were performed on dog-bone (type 1BA) specimens in accordance with ISO 527-2 using a Quasar 10 testing machine (Galdabini, Varese, Italy), equipped with a 1 kN load cell and operated at a crosshead speed of 10 mm/min. Given the limited number of RG materials and dog-bone specimens, preliminary tests were conducted at a crosshead speed of 1 mm/min. It was later verified that increasing the speed to 10 mm/min did not lead to significant differences in the elastic modulus measured with the extensometer. Therefore, all mechanical tests were ultimately carried out at a crosshead speed of 10 mm/min.

Charpy impact tests were conducted following ISO 179-1 [1] on notched specimens measuring 80 mm × 10 mm × 4 mm, with a 45° V-notch (2 mm depth) centered on the specimen. Testing was carried out using an AMSE HIT 2492 Series impact tester (Torino, Italy) equipped with a 2 J pendulum. For both mechanical tests, a minimum of five replicates per sample was analyzed to ensure statistical reliability.

To assess the suitability of the PP/RG composites for applications such as plant pot production, an area where polypropylene is widely employed, a phytotoxicity test was conducted using *Lepidium sativum* seeds exposed to eluates derived from thermally treated RG powder, both without and with SA, as well as from PP20 and PP20S composite pellets.

RG, SA-treated RG, PP20, and PP20S pellets were immersed in distilled water at a ratio of 1:10 g/mL for 24 h under magnetic stirring at 300 rpm. Subsequently, the solutions were centrifuged at 180 rpm for 2.5 min and then passed through a vacuum filter to remove the smallest RG particles. Three Petri dishes of 90 mm in diameter were prepared, each containing a Whatman 597 filter paper disc and 10 seeds of *Lepidium sativum.* In the three dishes, 2.5 mL of pure eluate (100%), 2.5 mL of eluate diluted with 50% distilled water (50%), and 2.5 mL of distilled water (0%) were added, respectively. Three replicates were performed for each sample. The dishes were incubated in the dark at 26 °C for 72 h. After incubation, germinated seeds were counted, and primary root length was measured to calculate the germination index (GI) according to Equation (2):

(2) $$GI\left(\%\right)=\frac{G_{c}\cdot L_{c}}{G_{t}\cdot L_{t}}\cdot 100$$

where G_c_ and G_t_ represent the number of germinated seeds in the sample and in deionized water, respectively, while L_c_ and L_t_ denote the mean root length in the sample eluate and in deionized water, respectively. The material is considered non-phytotoxic if the GI exceeds 80% [22].

Finally, the composite formulation, optimized in terms of processability and mechanical performance, was subsequently processed by injection molding to produce plant pots to confirm its suitability with industrial-scale manufacturing.

## 3. Results and Discussion

### 3.1. Thermal Analysis

To determine the appropriate temperature for the pretreatment of as-received gypsum aimed at obtaining its anhydrous form (CaSO_4_), the ground and sieved raw gypsum was first dried in an oven at 105 °C for 24 h to remove moisture, resulting in a weight loss of 22.5 wt.%. The dried gypsum was then heated at 200 °C for 24 h to promote the transformation from the dihydrate form (CaSO_4_·2H_2_O) to the anhydrous form (CaSO_4_) [23], leading to an additional weight loss of 16.7 wt.% relative to the starting material. This value is consistent with the expected content of CaSO_4_·2H_2_O originally present in the red gypsum (Table 1). Subsequently, RG was heated at 220 °C for an additional 24 h; however, no significant further weight loss was observed. In general, the dehydration of gypsum from the dihydrate to the anhydrite phase proceeds slowly. Therefore, the thermal treatment at 200 °C for 24 h was considered adequate to obtain the anhydrous form of CaSO_4_, and to produce a powder for processing in the extruder with PP.

**Figure 1:**
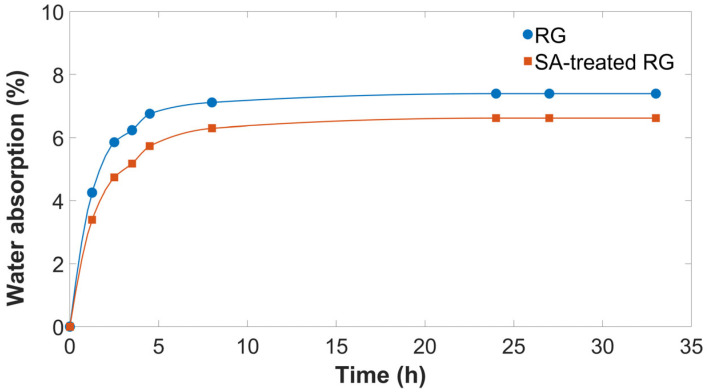
Moisture absorption of thermally treated RG with and without SA.

Furthermore, to evaluate the air stability of RG, both with and without SA treatment, water absorption measurements were performed by exposing the samples to an atmosphere with 70% relative humidity at 25 °C, using an environmental test chamber, and monitoring the percentage weight change over time (Figure 1). The test was conducted on a sufficiently large sample volume (200 g), considered representative of the overall morphology and characteristics.

As shown, both RG and SA-treated RG rapidly absorb moisture within the first 5 h, after which a plateau is reached. The sample without SA reaches a moisture content of 7.4 wt.%, whereas the SA-treated sample stabilized at 6.6 wt.%. This lower equilibrium moisture content can be attributed to the hydrophobic nature of stearic acid, which forms a partial surface barrier that hinders moisture diffusion [24]. Consequently, stearic acid not only acts as a compatibilizer between gypsum and the polymer matrix but also reduces atmospheric water uptake, an additional benefit that enhances material processability during extrusion.

**Figure 2:**
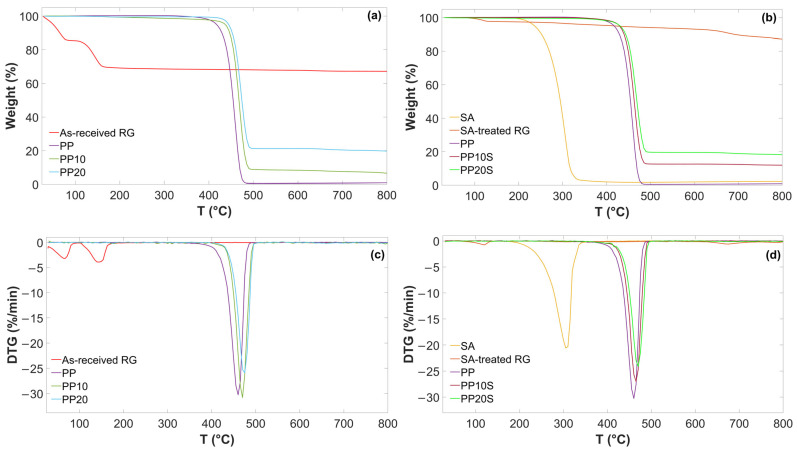
TG and DTG curves of (**a**,**c**) as-received RG, PP, PP10, and PP20; (**b**,**d**) SA, SA-treated RG, PP, PP10S, and PP20S.

TG and DTG curves of as-received RG, SA-treated RG, and the produced PP/RG composites are reported in Figure 2. The main thermal degradation temperatures (onset and peak temperatures) and relative weight losses are presented in Table 6.

As shown, two main weight loss steps are observed for the as-received RG. The first step, accounting for 14.1 wt.%, is attributed to the evaporation of residual moisture. The second step, amounting to 16.4 wt.% and characterized by a distinct peak, is associated with sequential dehydration reaction—from CaSO_4_·2H_2_O to CaSO_4_·^1^/_2_H_2_O, and subsequently to CaSO_4_—as well as with the thermal decomposition of iron, magnesium, and aluminum hydroxides into their corresponding oxides. These decomposition processes typically occur in the 100–200 °C temperature range. The observed weight losses are in good agreement with those recorded during oven-based thermal treatment previously described and are consistent with the composition presented in Table 1, particularly the contents of CaSO_4_·2H_2_O and metal hydroxides.

A sharp thermal degradation step is observed for SA, indicating that this fatty acid decomposes in one stage, with a T_onset_ of 272.7 °C and a final residue of 2.6 wt.% attributed to fixed carbon formed during pyrolysis [25]. Furthermore, SA-treated RG showed no weight loss in the 100–200 °C range and only a minor loss below 100 °C, attributable to residual moisture evaporation. This confirms that the anhydrite and iron/magnesium/aluminum oxides formed during thermal treatment at 200 °C did not reconvert to CaSO_4_·2H_2_O, or to the corresponding hydroxides upon subsequent exposure to air.

These findings confirm the thermal stability of both RG and SA up to at least 250 °C, supporting their suitability for compounding with the PP matrix at typical extrusion temperatures below this threshold (Table 4). As a result, neither significant water vapor release nor SA decomposition is expected during the extrusion process.

The addition of RG or SA-treated RG to the PP matrix delayed the degradation of PP, increasing both the onset and peak weight loss temperatures of the polymer matrix by 10–12 °C. This increase in the thermal stability of the composites is attributed to the presence of RG, which is believed to act as a barrier, hindering the release of volatile degradation products during thermal treatment and thereby slowing down polymer decomposition [26]. As expected, the addition of red gypsum filler to the PP matrix, with or without SA, also affects the final weight residue, increasing it from 1.0 wt.% for neat PP to 19.9 wt.% for the PP20 formulation, corresponding to the inorganic filler content.

**Figure 3:**
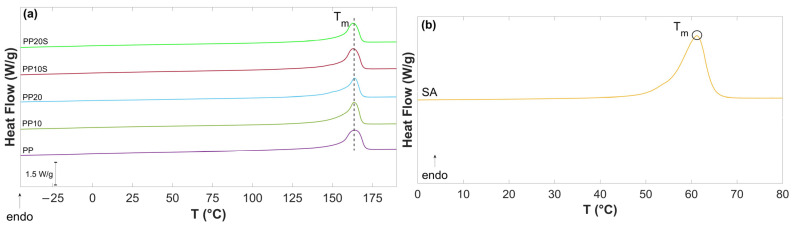
DSC thermograms of (**a**) PP, PP/RG composites, and (**b**) SA.

Figure 3 presents the second DSC heating curves of PP, PP/RG composites, and SA. The values of T_m_, ΔH_m_, and Χ_C_, calculated based on Equation (1), are reported in Table 7.

The thermograms show minor differences in the main thermal parameter (T_m_ and X_C_) which can be attributed to the filler–matrix interactions and the role of SA. The endothermic melting peak of SA observed in Figure 3b (T_m_ = 62.3 °C) is consistent with the value reported in the technical data sheet and aligns with the blending conditions described in Section 2.2.1, which enable the dispersion of SA with RG particles. Notably, this peak is absent in the PP/RG thermograms (Figure 3a), as RG is compatible with PP and acts as a plasticizer. This is supported by the slight reduction in both melting temperature and degree of crystallinity in the RG composites treated with SA (from T_m_ = 164 °C to 163 °C, and from Xc = 47.6% to 45.9% and 45.6%).

PP exhibits a crystallization temperature (Tc) of 110.7 °C. The incorporation of RG promotes crystallization by providing heterogeneous nucleation sites, resulting in an increase in Tc to 120.5 °C and 121.3 °C for PP10 and PP20, respectively. The compatibilization effect of SA (Tc = 46 °C) is also evident from the cooling curves of PP10S and PP20S, which do not display the SA crystallization peak and show lower Tc values (114.9 °C and 115.6 °C, respectively) compared to their non-compatibilized counterparts.

### 3.2. FTIR Analysis

**Figure 4:**
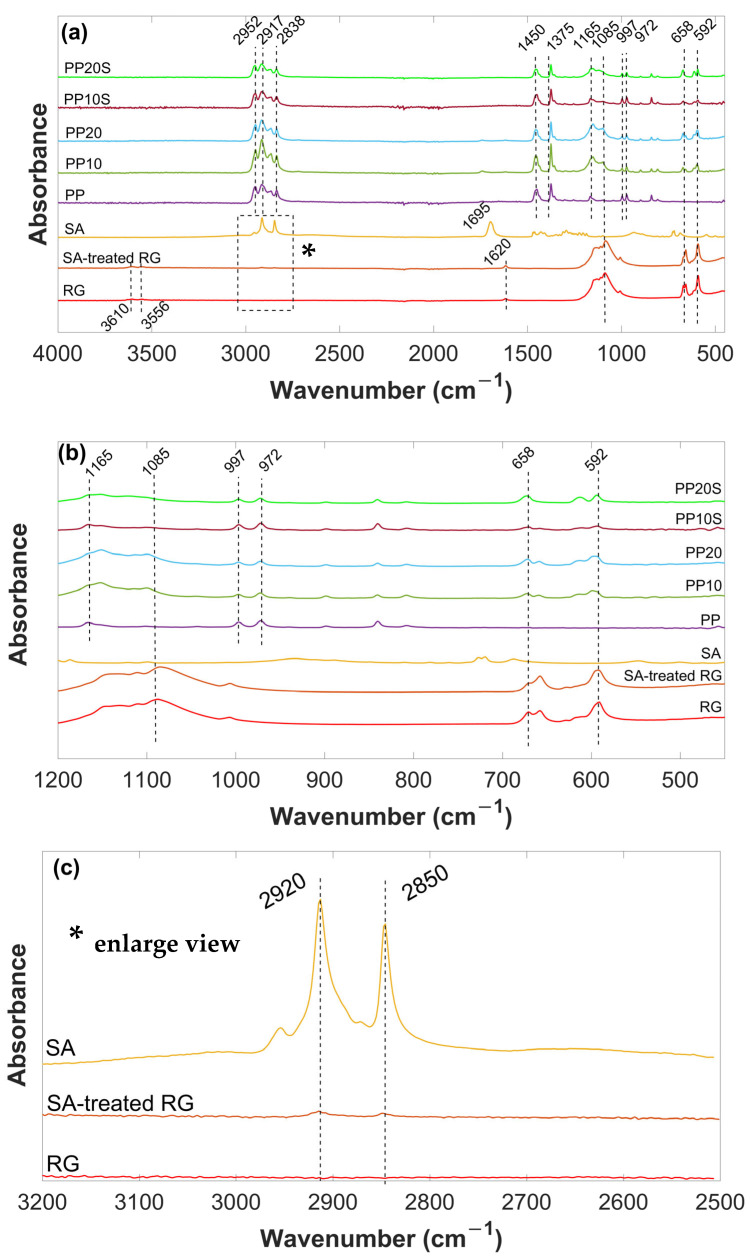
(**a**) FTIR spectra of SA, thermally treated RG (with and without SA), PP, and PP/RG composites in the range of 4000–450 cm^−1^; (**b**) FTIR spectra of SA, thermally treated RG (with and without SA), PP, and PP/RG composites in the range of 1200–450 cm^−1^; (**c**) enlarged view (*) of the FTIR spectra of SA and thermally treated RG (with and without SA) in the range of 3200–2500 cm^−1^.

FTIR spectra of RG, SA-treated RG, SA, PP, PP10, PP20, PP10S, and PP20S are reported in Figure 4. RG exhibits characteristic absorption bands in the IR spectrum corresponding to sulfate groups (SO_4_^2−^) at 1085 cm^−1^, 658 cm^−1^, and 592 cm^−1^; surface hydroxyl groups (OH) at 3556 cm^−1^ and 3610 cm^−1^; and crystal water (H_2_O) at 1620 cm^−1^. The presence of this latter small peak suggests the possible rapid surface formation of hemihydrate sulfate upon atmospheric exposure of the dehydrated material [27].

The SA, a saturated fatty acid with a long chain of 18 carbon atoms, is characterized by the adsorption peaks in the high-frequency region at about 2920 and 2850 cm^−1^, attributed to -CH_2_- asymmetric and symmetric stretching vibrations, respectively. In the low-frequency region, the peak at 1695 cm^−1^ is attributed to -COOH group. Additionally, the other weak peaks correspond to the skeletal C-C vibrations of the main chain [28].

After surface modification with SA, the characteristic peaks of SA at 2920 and 2850 cm^−1^, appeared in the spectrum of the SA-treated RG, indicating that SA was physically absorbed onto its surface [29]. The low intensity of these peaks is attributed to the small amount of stearic acid present in the RG (3 wt.%).

In the FTIR spectrum of PP, the absorption peak at 840 cm^−1^ is attributed to the -CH2 rocking vibration. The peaks at 972, 997, and 1165 cm^−1^ correspond to skeletal C-C vibrations and CH_3_ rocking. The symmetric bending vibration of the -CH_3_ group appears at 1375 cm^−1^, while the peak at 2952 cm^−1^ is assigned to the asymmetric stretching of -CH_3_. All these absorption bands are associated with the presence of methyl groups in PP. Additionally, the peaks at 1450, 2838, and 2917 cm^−1^ are ascribed to the symmetric bending, symmetric stretching, and asymmetric stretching vibrations of -CH_2_- groups, respectively [30,31,32].

The spectra of the PP/RG composites without stearic acid (PP10 and PP20) display the characteristic peaks of both PP and RG, with no evident shifts in their positions, indicating the absence of chemical interactions between RG and the PP polymer matrix. Given the small amount of SA present in the PP10S and PP20S composites (0.3 and 0.6 wt.%, respectively), its characteristic peaks are not detectable in their spectra. Hence, the spectra of PP10S and PP20S closely overlap with those of PP10 and PP20.

### 3.3. Mechanical Analysis

**Figure 5:**
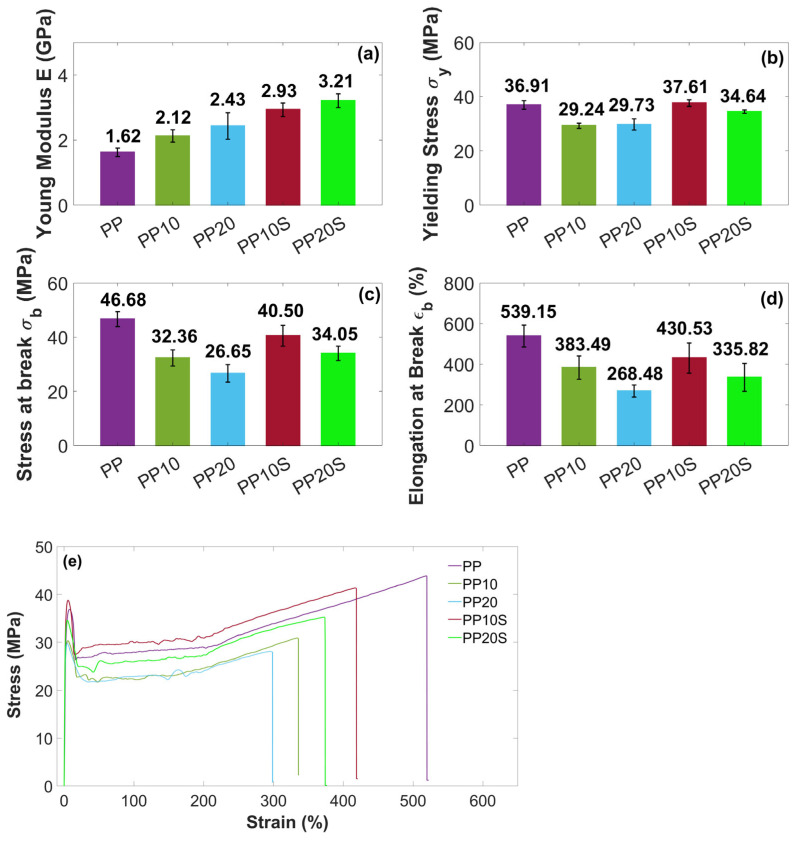
Tensile test results of (**a**) Young’s modulus E, (**b**) yielding stress σ_y_, (**c**) stress at break σ_b_, and (**d**) elongation at break ε_b_ of PP and PP/RG composites. Mean values ± standard deviation based on 10 replicates; (**e**) representative stress–strain curves.

Figure 5 presents the mean values and the corresponding standard deviations of the tensile properties of the PP/RG composites compared to those of the neat PP.

Under tensile loading, all composites showed the typical ductile behavior of an isotactic semicrystalline PP, characterized by an initial yielding followed by pronounced necking, high elongation at break (up to about 540%), and subsequent strain hardening due to molecular chain alignment along the stretching direction [33,34].

With the addition of RG, both with and without SA, the elastic modulus increased from 1.62 GPa (PP) up to 3.21 GPa (PP20S). This is associated with an embrittling effect, and consequently with a reduction in stress at break and elongation at break. These behaviors can be attributed to the filler’s ability to restrict the mobility of polymer chains, thereby stiffening the composite. In contrast, the reductions in stress at break and elongation at break are ascribed to a weak matrix–filler interface, which limits stress transfer between the two phases. Additionally, the filler particles may act as stress concentrators, promoting premature failure and consequently lowering both stress at break and elongation at break. This behavior has been reported in several studies [35,36,37], where composites with poor interfacial interactions between the filler and polymer matrix exhibited higher elastic modulus but reduced stress at break compared to the neat polymer.

Notably, at equal RG filler content, the presence of SA generally results in higher elastic moduli and stresses at break, which can be attributed to improved interfacial adhesion between the matrix and the filler [16], as demonstrated by previous DSC thermal analysis. Moreover, SA may enhance the dispersion of RG within the PP matrix, reducing the formation of RG agglomerates and stress concentration points, thereby improving ductility. In fact, at the same filler content, samples PP10S and PP20S exhibit higher elongation at break compared to PP10 and PP20, respectively.

These results highlight the positive effect of adding even small amounts of SA (up to 0.6 wt.%) on the mechanical properties of the PP/RG composites. Specifically, the presence of SA helps to limit the reduction in stress at break of PP to 13% and 31% for composites containing 10 wt.% and 20 wt.% RG, respectively, compared to reductions of 30% and 43% observed in the absence of SA.

**Figure 6:**
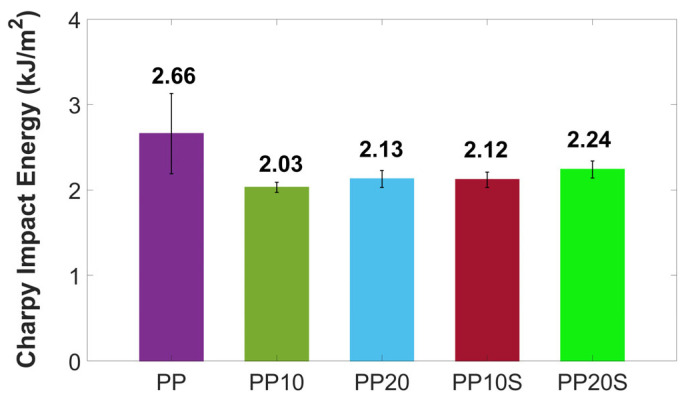
Charpy impact energy of PP and PP/RG composites. Mean values ± standard deviation based on 10 replicates.

The mean impact strength values of the PP/RG composites, compared to those of neat PP, are presented in Figure 6. As shown, the addition of red gypsum reduces the impact strength of PP-based composites due to increased brittleness, as also observed in the tensile tests, and a diminished ability to dissipate stress caused by discontinuities introduced into the polymer matrix. These effects are partially mitigated by improved filler dispersion and enhanced matrix–filler adhesion promoted by the presence of SA. On average, a reduction of 20–24% in Charpy impact energy is observed for the PP20 and PP10 composites, compared to a lower reduction of 16–20% for the PP20S and PP10S composites, respectively.

Considering that neat PP exhibits a mean Charpy impact energy of 2.66 kJ/m^2^ and is commonly used also for plant pots, the slightly lower impact values of 2.03–2.24 kJ/m^2^ observed in the PP/RG composites remain suitable for such applications, as explored in this study.

### 3.4. Morphological Analysis

A morphological analysis was conducted to evaluate the particle size distribution of RG and SA-treated RG, as well as their dispersion within the PP matrix.

**Figure 7:**
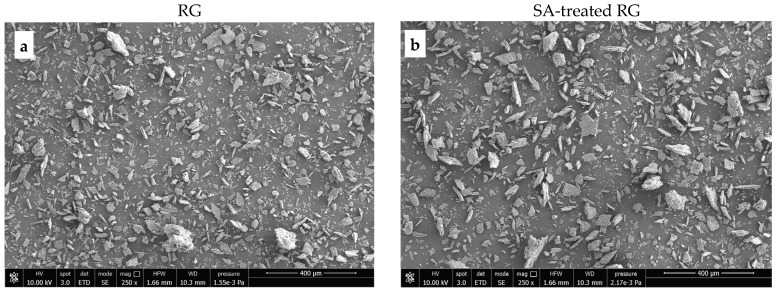
SEM images of (**a**) thermally treated RG; (**b**) SA-treated RG. Magnification ×250.

**Figure 8:**
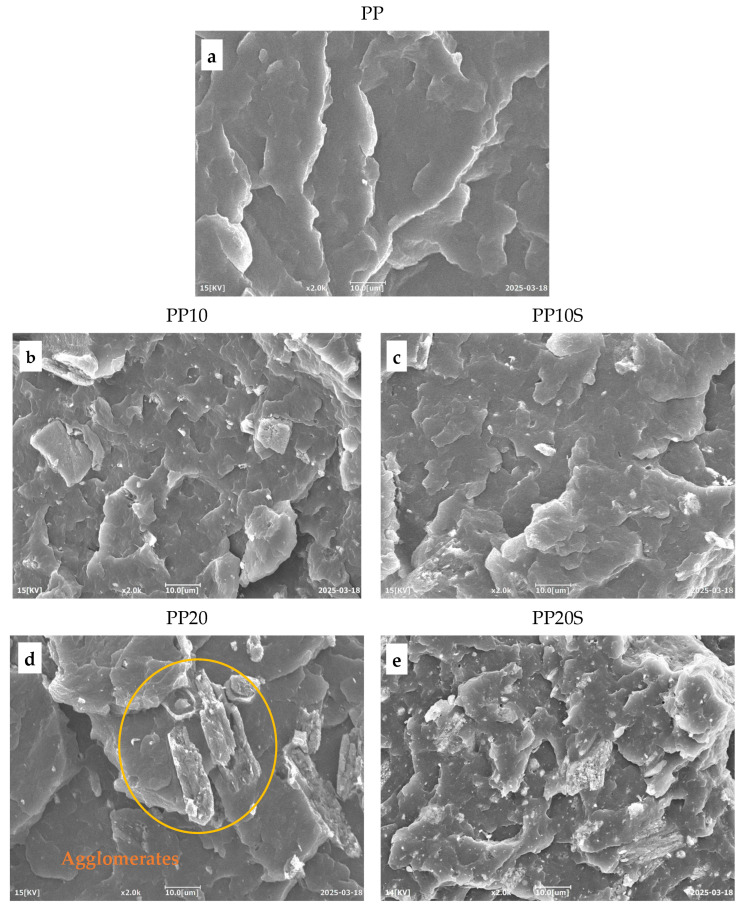
SEM images of PP/RG composites: (**a**) PP, (**b**) PP10, (**c**) PP10S, (**d**) PP20, and (**e**) PP20S. Magnification ×2000.

Figure 7 and Figure 8 show SEM images of the starting materials and the fracture surfaces of dog-bone specimens of the PP/RG composites, respectively. As shown in Figure 7, the red gypsum powder—after thermal treatment, pulverization, and sieving—with or without the addition of SA—exhibits a relatively uniform range of particle sizes, all below 100 microns.

The fracture surfaces of the PP/RG composites do not show any visible voids, confirming that no moisture or gas release occurred during extrusion within the 170–230 °C temperature range (Table 4). This also confirms the effective conversion of CaSO_4_·2H_2_O into CaSO_4_ through the preliminary thermal treatment of red gypsum.

In both composite series, with and without SA, the filler particles were discernibly distributed throughout the polymer matrix. Notably, in the presence of SA, a finer particle morphology is observed. This is particularly evident when comparing Figure 8d (PP20), which reveals the presence of agglomerates, with Figure 8e (PP20S), which demonstrates a more uniform particle size distribution. These results indicate that SA contributes to improved filler dispersion within the matrix and supports the development of structurally more homogeneous composites [16]. This trend aligns with the findings from the previously conducted mechanical characterization, which show an increase in elastic modulus with rising filler and SA contents (Figure 5a). The presence of agglomerates acts as preferential sites for crack initiation, making the material more susceptible to failure, and therefore less resistant and less ductile. This is supported by lower stress at break and elongation at break values recorded for PP10 and PP20 compared to their counterparts, PP10S and PP20S (Figure 5c,d). Likewise, the improved filler distribution enhances the material’s impact resistance, as evidenced by the higher Charpy impact energy values measured for PP10S and PP20S compared to the corresponding formulations without stearic acid (Figure 6).

### 3.5. Phytotoxicity Test

**Figure 9:**
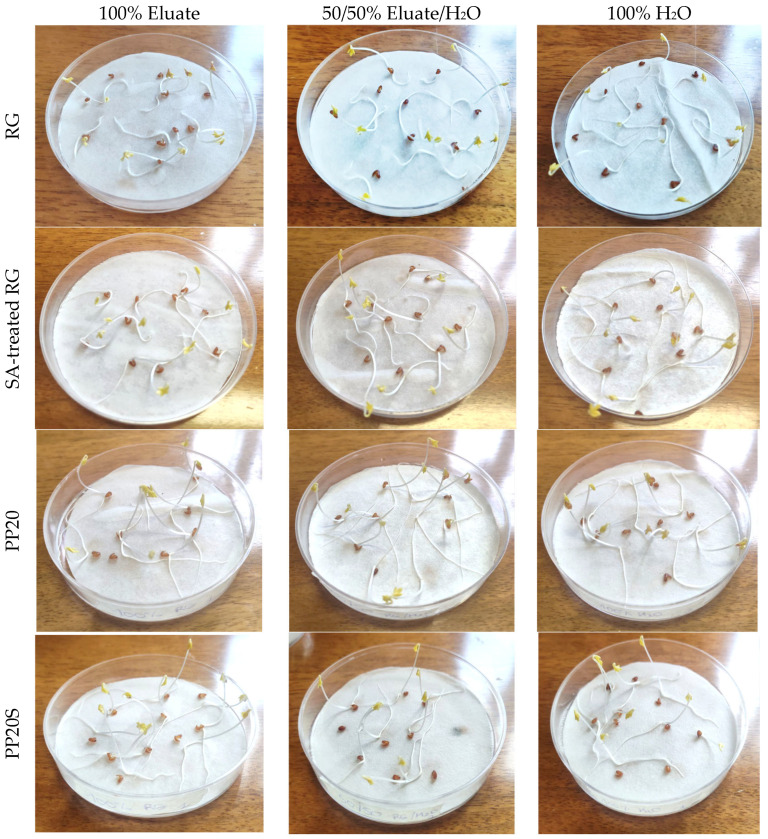
Petri dishes in the case of 100% eluate, 50% eluate, and 100% distilled water for RG, SA-treated RG, PP20, and PP20S pellets.

In view of the potential use of RG in PP-based composites for the production of agricultural items such as plant pots, a preliminary phytotoxicity test was conducted using eluates obtained from RG powder, with and without the addition of SA, as well as from PP/RG composite pellets with higher RG content (PP20 and PP20S). As an example, Figure 9 shows Petri dishes containing germinated seeds after 72 h of exposure to 100% and 50% RG eluates, along with 100% distilled water.

Table 8 reports the germination indices (GI) of *Lepidium sativum* seeds obtained at the end of the incubation period. As shown, the GI values for the composite pellets (PP20 and PP20S), calculated according to Equation (2), are well above the threshold value of 80%, considered safe for eluates obtained from standard compost [22], and are approximately equal or higher than those obtained with distilled water (control sample).

While the GI values of both RG and SA-treated RG eluates are below 80%, indicating phytotoxic effects on the germination of *L. sativum* seeds, a significant reduction in root length was also observed compared to seeds germinated in 100% distilled water. This effect may be attributed to the pH of the eluates and the presence of specific dissolved elements or metals [38,39,40]. To better understand the factors contributing to the phytotoxicity of the RG eluates, the chemical composition of the 100% eluate, obtained by mixing RG with distilled water at a 1 g:10 mL ratio for 24 h, was analyzed and is reported in Table 9.

Based on the chemical analysis of the leachate, the phytotoxicity observed in the sample derived from red gypsum is primarily attributed to the high concentration of sulfate ions (2180 mg/L), rather than to heavy metals such as Pb, Hg, Cu, Cd, etc., whose concentrations were all well below the levels considered phytotoxic [41]. These unbound sulfate ions may interact with essential nutrient ions, such as Zn and Mg, potentially reducing their bioavailability and inhibiting root development [42].

However, in the composites, the gypsum remains embedded within the PP matrix, thereby significantly limiting or preventing the release of sulfate ions into aqueous media. This is confirmed by the higher germination indices observed when using eluates from PP20 and PP20S pellets compared to RG and SA-treated RG. Therefore, the developed composites appear suitable for use in agricultural applications without inducing phytotoxic effects.

### 3.6. Injection-Molded Pot Production

Based on the favorable results obtained regarding processability, mechanical properties, and phytotoxicity, PP10S pellets were selected to produce plant pots using an industrial injection molding press (Canbio s180TH670, Negri Bossi, Milano, Italy) at Lippert (Florence, Italy). Processing parameters were set to a screw speed of 190 rpm and a back pressure of 6 bar, with the temperature profile detailed in Table 10.

**Figure 10:**
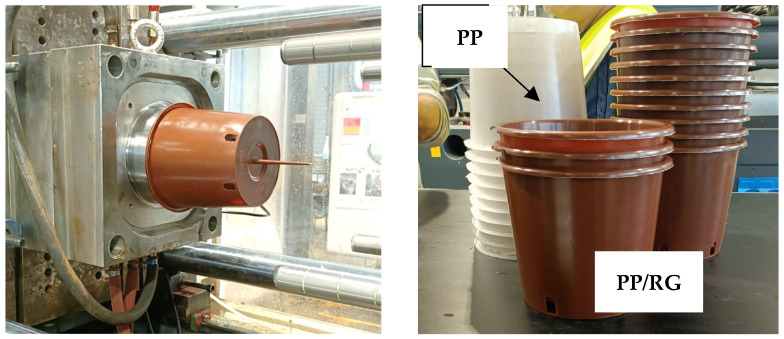
Pots produced with PP10S pellets by industrial injection molding.

Figure 10 shows some photos of the PP10S molded pots for agricultural use. No issues were encountered during the molding process.

As shown by the comparison between pots made of neat PP and those made of the PP10S composite, the addition of 10 wt.% RG imparts a brick-red coloration to the pots, confirming its role as a pigment filler.

## 4. Conclusions

In this study, polypropylene (PP)-based composites containing red gypsum (RG), a by-product of the titanium dioxide (TiO_2_) production process, were prepared via melt extrusion and subsequently processed by injection molding. Prior to compounding, RG was thermally treated at 200 °C to promote the removal of moisture and chemically bound water, thereby yielding its anhydrous form (CaSO_4_), which is more suitable for processing with PP. The incorporation of stearic acid (3 wt.% relative to RG) enhanced filler dispersion within the polymer matrix by reducing its hygroscopicity and lowering friction during extrusion.

Thermal analysis revealed that the addition of RG did not significantly affect the melting temperature or the degree of crystallinity of the polymer compared to neat PP. A slight increase in thermal degradation temperature was observed, suggesting a stabilizing effect imparted by RG.

Mechanical testing confirmed that stearic acid played a key role in enhancing filler dispersion and strengthening filler–matrix adhesion, resulting in an increase in Young’s modulus from 1.62 GPa (neat PP) to 3.21 GPa with 20 wt.% RG and 0.6 wt.% SA. The use of SA mitigates the typical stiffening effect associated with the incorporation of inorganic fillers, which generally reduce stress at break and elongation at break. All composites showed a moderate reduction in impact strength with increasing RG content, ranging from 2 to 2.12 kJ/m^2^ for RG loadings up to 20 wt.%, compared to 2.66 kJ/m^2^ for neat PP. Nevertheless, these values remain acceptable for moderate-load applications, such as those considered in the present study.

Preliminary phytotoxicity tests using *Lepidium sativum* L. seeds showed no phytotoxic effects of the eluates of PP/RG composite pellets, confirming their potential for safe use in agricultural contexts.

Finally, the good processability of these composites by both extrusion and injection molding, even at RG contents as high as 20 wt.%, demonstrates that this industrial by-product can be effectively reused as a pigmenting filler. RG thus represents a sustainable alternative to iron-based pigments and offers a viable route for the valorization of TiO_2_ industry waste in practical applications, particularly in the floriculture and horticulture sectors, where PP is widely used.

## Figures and Tables

**Table 1:** Chemical composition of filter-pressed red gypsum by Venator Sr, determined following the method UNI EN 15309:2007 (on dry basis).

Component	wt.%	Component	mg/kg
SO_3_	40.8	Zn	15
CaO	30.2	Co	9.3
CO_2_	2.29	Ni	9.2
Fe_2_O_3_	4.35	Cu	6.5
SiO_2_	1.60	Pb	3.5
TiO_2_	1.47	As	1.8
MgO	1.09	Cd	0.2
Al_2_O_3_	0.97	Cr (IV)	<0.2
MnO	0.13	Hg	0.05
V	0.08	Other metal traces	
Cr	0.03		
Loss at 200 °C (H_2_O bound)	19.4		

**Table 2:** Properties of PP Moplen HP500N^®^.

Properties	Value
Melt Flow Rate (230 °C/2.16 Kg)	12 g/min
Density	0.9 g/cm^3^
Young’s Modulus	1.55 GPa
Yielding Stress	34 MPa
Elongation at Break	>500%

**Table 3:** Compositions of the produced PP/RG composites.

Sample	PP (wt.%)	RG (wt.%)	SA (wt.%)
PP	100.0	0	0
PP10	90.0	10	0
PP20	80.0	20	0
PP10S	89.7	10	0.3
PP20S	79.4	20	0.6

**Table 4:** Extruder temperature profile for the composite preparation.

Feeding Zone				Extruder Head
50 ± 1 °C	170 ± 1 °C	215 ± 1 °C	230 ± 1 °C	210 ± 1 °C

**Table 5:** Temperature profile of the injection press for dog-bone and Charpy specimens.

Hopper				Nozzle & Mold
55 ± 1 °C	200 ± 1 °C	205 ± 1 °C	205 ± 1 °C	215 ± 1 °C

**Table 6:** Degradation temperatures and corresponding percentages of weight loss and residue. * Calculated as the asymptotic value at 800 °C. ** Calculated at the extrapolated temperature corresponding to the onset of weight loss.

Sample	T_onset_ (°C)		T_d_ (°C)		Residue (wt.%) *	Weight Loss (wt.%) **	
As-received RG	51.7	122.8	76.4	143.5	67.8	14.1	16.4
SA-treated RG	99.4	647.1	120.0	674.2	87.3	4.5	3.9
SA	272.7		307.0		2.3	97.6	
PP	438.2		458.3		1.0	99.6	
PP10	451.8		470.1		7.5	91.2	
PP20	455.1		473.1		19.9	78.9	
PP10S	444.9		463.0		12.6	87.2	
PP20S	449.1		469.0		19.6	79.8	

**Table 7:** Tm, ΔHm and Χc of the DSC tested samples.

Sample	T_m_ (°C)	ΔH_m_ (J/g)	Χ_C_ (%)
SA	62.3	184.4	-
PP	164.0	98.6	47.6
PP10	163.8	89.3	47.9
PP20	163.8	78.0	47.1
PP10S	163.0	85.5	45.9
PP20S	163.0	75.5	45.6

**Table 8:** Germination Index of 50/50 and 100% eluates.

Sample	50/50 Eluate/H_2_O	100% Eluate
RG	47.9 ± 5.2	41.5 ± 7.8
SA-treated RG	50.4 ± 14.6	47.6 ± 7.7
PP20	143.3 ± 45.9	98.1 ± 20.3
PP20S	110.6 ± 12.2	124.6 ± 26.3

**Table 9:** pH and chemical composition (mg/L) of eluate of dried RG.

Parameter	Value	Parameter	Value
pH	7.75	Ni *	0.00136 ± 4.0·10^−4^
As *, Be *	<0.0001	Zn *	0.0161 ± 8.4·10^−3^
Pb *, Se *	<0.001	Co *	0.00029 ± 1.2·10^−4^
CN− **	<0.01	V *	0.0028 ± 1.5·10^−3^
Ba *	0.0115 ± 0.0073	Cl− ***	241 ± 48
Cd *	0.000116 ± 3.9·10^−5^	$F^{-}$ ***	0.258 ± 0.049
Cr (tot) *	0.0011 ± 5.8·10^−4^	${SO}_{4}^{2-}$ ***	2180 ± 480
Cu *	0.00159 ± 7.2·10^−4^	${NO}_{3}^{-}$ ***	3.06 ± 0.58
Hg *	5.2 10^−5^ ± 2.7·10^−5^		

* UNI EN 12457-2:2004 + UNI EN ISO 17294-2:2016; ** UNI 10802:2013 Appendix A.2 + UNI EN 12457-2:2004 + M.I. 2088 rev 0 2006; *** UNI EN 12457-2:2004 + UNI EN ISO 10304-1:2009.

**Table 10:** Thermal profile of injection press for PP10S pots.

Hopper			Nozzle & Mold
185 ± 1 °C	185 ± 1 °C	182 ± 1 °C	180 ± 1 °C

## Data Availability

The original contributions presented in this study are included in the article. Further inquiries can be directed to the corresponding author(s).
